# The Intricate Interplay between Cancer Stem Cells and Oncogenic miRNAs in Breast Cancer Progression and Metastasis

**DOI:** 10.3390/life13061361

**Published:** 2023-06-09

**Authors:** Antonis Tsintarakis, Chara Papalouka, Christina Kontarini, Panagiotis Zoumpourlis, Konstantinos Karakostis, Maria Adamaki, Vassilis Zoumpourlis

**Affiliations:** 1 Biomedical Applications Unit, Institute of Chemical Biology, National Hellenic Research Foundation (NHRF), 11635 Athens, Greece; 2 Institut de Biotecnologia i de Biomedicina, Universitat Autònoma de Barcelona, 08193 Barcelona, Spain

**Keywords:** breast cancer, BCSCs, miRNAs, stemness, EMT, metastasis, precision medicine

## Abstract

Complex signaling interactions between cancer cells and their microenvironments drive the clonal selection of cancer cells. Opposing forces of antitumor and tumorigenic potential regulate the survival of the fittest clones, while key genetic and epigenetic alterations in healthy cells force them to transform, overcome cell senescence, and proliferate in an uncontrolled manner. Both clinical samples and cancer cell lines provide researchers with an insight into the complex structure and hierarchy of cancer. Intratumor heterogeneity allows for multiple cancer cell subpopulations to simultaneously coexist within tumors. One category of these cancer cell subpopulations is cancer stem cells (CSCs), which possess stem-like characteristics and are not easily detectable. In the case of breast cancer, which is the most prevalent cancer type among females, such subpopulations of cells have been isolated and characterized via specific stem cell markers. These stem-like cells, known as breast cancer stem cells (BCSCs), have been linked to major events during tumorigenesis including invasion, metastasis and patient relapse following conventional therapies. Complex signaling circuitries seem to regulate the stemness and phenotypic plasticity of BCSCs along with their differentiation, evasion of immunosurveillance, invasiveness and metastatic potential. Within these complex circuitries, new key players begin to arise, with one of them being a category of small non-coding RNAs, known as miRNAs. Here, we review the importance of oncogenic miRNAs in the regulation of CSCs during breast cancer formation, promotion and metastasis, in order to highlight their anticipated usage as diagnostic and prognostic tools in the context of patient stratification and precision medicine.

## 1. Introduction

Breast cancer (BC) is a multifactorial disease whereby normal cells accumulate mutations, transform and lose their replicative control. The latest statistics show that breast cancer is becoming a major cause of death, accounting for more than 2.3 million out of 10 million deaths from cancer worldwide [1,2]. Germline and somatic mutations are major contributors to the malignant transformation of normal cells and cancer development [3,4]. Apart from their mutational burden, cells are constantly exposed to factors that alter their epigenetic profile, leading to malignancy or contributing to a more aggressive phenotype of already transformed cells [4].

Although breast cancer is a highly heterogenous disease, specific immunohistochemical (IHC) markers have been identified, dividing BC into different subgroups. These IHC markers include the estrogen receptor (ER), progesterone receptor (PR) and human epidermal growth factor receptor 2 (HER2). The simultaneous absence of ER, PR, and HER2 is defined as triple-negative BC (TNBC) [5]. Further gene expression analysis led to the classification of BC into five molecular subgroups: luminal A tumors, which constitute the most common type of breast cancer; luminal B tumors; human epidermal growth factor receptor-2 (HER2)-enriched tumors; basal-like tumors; and normal-like BC [5,6,7]. The basal-like subtype is a triple-negative aggressive BC with a poor clinical outcome, consisting of a great proportion of breast cancer stem cells and characterized by the most common BCSC biomarkers, CD44^+^/CD24^−/low^ and ALDH1^+^ [5].

Breast cancer stem cells (BCSCs) are a subpopulation of undifferentiated breast cancer cells, originally identified and isolated in 2003 by Al-Hajj et al. [8], which maintain both normal stem-cell and cancer-cell features, such as self-renewal and differentiation capabilities, altered gene expression profiles and highly tumorigenic properties, giving rise to the heterogeneity of breast tumors [9,10]. BCSCs are also implicated in tumor progression, epithelial-to-mesenchymal transition (EMT), invasion, metastasis, resistance to therapy and recurrence [9,10]. To date, the most commonly used biomarkers of BCSCs are the cell surface markers, CD44^+^/CD24^−/low^, the aldehyde dehyogenase 1 (ALDH^+^) and the epithelial specific antigen (ESA), also known as CD326 or EpCAM [5,10]. A number of studies report less frequently used biomarkers, such as ABCG2, CD133, CD49f, LGR5, SSEA-3, CD70, PROCR, MUC1 (EMA), GD2, Nectin-4 and CD90 [5,10].

A widely known stem-like property of breast cancer cells (BCCs) is the formation of mammospheres, i.e., distinct clusters of cells in a serum-free culture. Sphere-forming ability is a common feature of CSCs, and Ponti et al. first observed that spheres generated from breast cancer cell lines and primary breast tumors consist of about 95% CD44^+^/CD24^−/low^ cells [11]. To date, mammosphere assays are widely used to identify the number of cells with self-renewal capability and the stemness potency of BCCs in vitro (mammosphere formation efficiency, *MFE*) in combination with other methodologies, such as the CD44^+^/CD24^−^ or ALDH^+^ cell ratio, gene expression profiling, the proliferation and migration rate, etc. [10,12,13,14].

In recent years, a significant number of research teams have focused on elucidating the role of small non-coding RNAs in the regulation of gene expression. In the case of microRNAs, during research in post-embryonic development of Caenorhabditis elegans, the first identified member of the family was *lin-4*, [15,16]. MicroRNAs are single-stranded RNAs (ssRNAs) consisting of about 20–25 nucleotides. They are generated by the Dicer endoribonuclease, which recognizes a unique hairpin secondary structure of the precursor molecules [17]. The corresponding genes of these precursors can be traced in intragenic regions and introns of messenger RNAs or within non-coding RNA genes [18,19]. Further research on miRNA-encoding genes revealed that more than one-third of known human miRNA genes appear in clusters of two or more, closely positioned to each other, located in the same DNA strand and transcribed together as polycistronic transcripts [20,21].

Several of the functions performed by miRNAs in human physiology and pathology have been identified, including signal transmission, molecular network stability maintenance, nervous system regulation, cell differentiation and development, reproduction and immunity. Current evidence highlights the pivotal role of miRNAs in the regulation of gene expression in carcinogenesis concerning tumor initiation, progression, resistance to therapy and metastasis [22]. In addition, potential onco-suppressor properties of miRNAs are being investigated, and promising miRNA mimic technology is under development with high prospects of being used in novel anti-cancer treatment strategies [23].

In this review, we summarize the latest findings regarding oncogenic miRNAs, alternatively named oncomiRs [23], and review the involvement of CSCs and stemness-related functions in different subtypes of BC. The miRNAs listed in the main part were carefully selected after the meticulous curation of studies obtained from the commercially available database “PubMed” utilizing a text mining approach. Terms such as “Cancer Stem Cells”, “Breast Cancer” and “miRNAs” were searched for in combination, and the abstracts from the resulting articles, published within the last ten years, were screened to determine their relevance with our search. The articles were then analyzed, and the information related to the interactions between BCSCs and oncogenic miRNAs was extracted and used for writing the main body of this review.

## 2. MiRNAs Exerting Oncogenic Functions in BCSCs and Stem-Like BCCs

**Figure 1:**
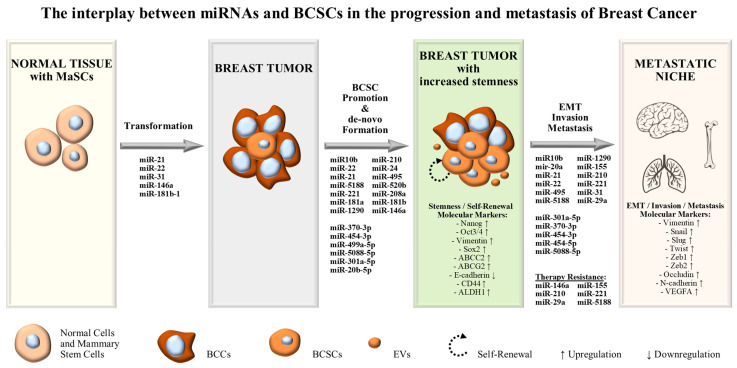
The interplay between miRNAs and BCSCs in the progression and metastasis of breast cancer.

The subgroup of oncogenic microRNAs or “oncomiRs” consist of microRNAs that function as oncogenes, promoting carcinogenesis, malignant transformation and metastasis. Usually, “oncomiRs” are up-regulated in tumors, inhibiting tumor suppressive genes. Currently, miRNA expression profiling is largely associated with tumor progression and tumor staging, and thus employed for predicting responses to treatment. In the context of BCSCs, “oncomiRs” may promote breast cancer stemness by increasing the number of stem-like cells in tumors, inducing mammosphere formation and contributing to EMT and metastasis (Figure 1) [24,25]. Moreover, as discussed above, breast cancer stem-like cells include a dynamic cell population that is regulated by a range of molecules in the tumor microenvironment. Theoretically, they are capable of unlimited self-renewal capacity and considered to be the major survivors of traditional chemo- and radio-therapies, leading to therapy-resistant tumors (Figure 1). In improvement of traditional anti-cancer treatments, aiming to reduce the tumor bulk by killing non-stem proliferating cells, the study of miRNAs, acting as molecular regulators that induce stemness in the tumor microenvironment, will bring a new era in breast cancer management [25].

### 2.1. miR-155

The miR-155 was found to be up-regulated in BC cells and BCSCs isolated from both human breast cancer samples and cell lines, and an increased content of miR-155 was also detected in exosomes derived from BCSCs and other chemo-resistant cells [26,27,28]. Higher expression levels of miR-155 in MDA-MB-231 cells were found to be positively associated with sphere growth, stem cell formation (ABCG2, CD44^+^/CD24^−/low^ and CD90^+^), the enhancement of stem-like properties of BCCs and chemoresistance to Doxorubicinol [27]. Moreover, miR-155 was detected to be enriched in exosomes derived from BCSCs and chemo-resistant cells, capable of targeting C/EBP-β, TGF-β and FOXO3a, subsequently enhancing the stem-like characteristics of MCF-7 and MDA-MB-231 cells, inducing EMT-related resistance to chemotherapies and increasing their migration potential [28].

### 2.2. miR-208a

A potential role for miR-208a in BC stemness has been proposed since its over-expression in a mammosphere model involving triple-negative MDA-MB-231 and ER^+^ ZR75-1 cell lines led to an increased proportion of ALDH1^+^ BCSCs [29]. The same researchers reported that miR-208 was capable of inducing SOX2 and β-catenin expression, stimulating LIN28 production and leading to the inhibition of a major tumor-suppressor miRNA, let-7a, thus inducing the BCSC phenotype. The restoration of let-7 significantly inhibited the stemness related to miR-208 over-expression, indicating the existence of a miR-208a-SOX2/β-catenin-LIN28-let-7a regulatory feedback loop [29].

### 2.3. miR-210

Camps et al. proposed miR-210 as an independent prognostic marker for the overall survival of BC, highlighting the link between miR-210 and hypoxia via the mediation of HIF1α/VHL transcriptional system [30]. Increased expression levels of miR-210 in MDA-MB 231 and MCF7 mammospheres after high doses of radiation were shown to stabilize hypoxia-inducible factor-1 and enhance radioresistance in vitro [31]. Likewise, miR-210 was found to be up-regulated in a BCSC-rich MCF7 spheroid model and could enhance migration, invasion, proliferation and self-renewal by directly targeting E-cadherin and causing the up-regulation of a known stemness marker and the E-cadherin repressor, Snail [32].

### 2.4. miR-24

MiR-24-3p was found to be up-regulated in both plasma and tissue samples from patients with metastatic BC, and miR-24 over-expression was associated with the metastatic process and a lower survival rate [33]. The up-regulation of miR-24 in BCSCs was also reported to promote mammosphere formation in the T47D, MCF-7 and MDA-MB-231 cell lines and induce adaptive response to hypoxia conditions by up-regulating HIF1α expression via directly targeting the HIF1α repressor, FIH1 [34]. Additionally, miR-24 up-regulated stemness genes, such as Nanog, Oct-3/4 and Vimentin, and down-regulated E-cadherin in T47D and MCF-7 cells. Finally, miR-24 could also induce resistance to cisplatin by directly targeting bimL and down-regulating its expression [34].

### 2.5. miR-20a and miR-20b

miR-20a and miR-20b are considered members of the miR-17 family, share a high sequence homology and identical seed sequences and are located on different chromosomes potentially due to evolutionary gene duplication events [35]. Both miRNAs have been implicated in BC, performing different functions depending on the context. A differential distribution between these two miRNAs has been reported and a potential correlation with the metastatic heterogeneity of BC was proposed, while evaluating primary tumors from patients with high/low invasive metastatic BC [36].

Guo et al. reported the over-expression of miR-20a-5p in clinical samples from BC patients and then showed that miR-20a-5p promotes the migration and invasion of MDA-MB-231 cells [37]. Furthermore, increased levels of miR-20a-5p were detected in MDA-MB-231-derived exosomes, capable of modulating the tumor microenvironment by targeting SRC kinase signaling inhibitor 1 (SRCIN1) in murine bone marrow macrophages (BMMs), and thus promoting their proliferation and osteoclastogenesis [37]. A mechanism of BCSC immunoevasion involving an miR-20a/MICA/MICB signaling axis has also been described [38]. The aberrant expression of miR-20a was capable of down-regulating two ligands of the stimulatory NK cell receptor (NKG2D), MICA and MICB, thus enhancing BCSC resistance to NK cell cytotoxicity and contributing to lung metastasis [38].

In addition, the tumorigenic effect of miR-20b-5p in both BC cells and BCSCs, leading to increased proliferation and survival of T47D-CSCs via the overall up-regulation of CCND1 and E2F1, was recently studied [39].

### 2.6. miR-10b

Ma et al. were among the first to describe the oncogenic functions of miR-10b in BC, detecting its over-expression in metastatic breast cancer cells [40]. Specifically, they reported that a known stemness-related factor, Twist, could regulate miR-10b expression and that miR-10b could regulate cell migration and invasion both in vitro and in vivo via the direct targeting of HOXD10, thus activating the pro-metastatic gene, RHOC [40]. A few years later, another study involving BCSCs from MCF-7, SKBR-3 and MDA-MB-231 cells indicated that miR-10b could promote self-renewal, stemness and EMT markers, such as OCT4/3, SOX2, SNAIL and Vimentin [41]. The same study group concluded that the underlying mechanism involved the direct targeting of PTEN by miR-10b, thus maintaining AKT pathway activation, events that could be negated by the inhibition of miR-10b expression.

### 2.7. miR-21

miR-21 is a frequently deregulated oncomiR in various types of solid tumors that has been established as an important oncogenic factor in BC and implicated in tumor progression, EMT regulation, CSC formation, invasion and metastasis [42,43]. While the mechanisms underlying miR-21 transcription regulation in BC have not been thoroughly elucidated, Chen and Bourguignon proposed a mechanism in which Hyaluronan and CD44 interaction induces c-Jun signaling and miR-21 expression, subsequently leading to BCL2 up-regulation and chemoresistance in the widely used triple-negative breast cancer cell line, MDA-MB-468 [44]. Additionally, Iliopoulos et al. highlighted the importance of miR-21 expression at the early stages of malignant transformation of non-tumorigenic cells, while experimenting on a non-transformed mammary epithelial cell line (MCF-10A) containing ER-Src [45]. In their model IL-6, an aberrantly expressed inflammatory cytokine, could induce STAT3 activation, an event critical for transformation. STAT3 directly activated miR-21, among other miRNAs, which directly targeted PTEN, leading to increased Akt activity and subsequent NF-κB activation, thus maintaining a transformed state. This transformed state of MCF-10A cells increased their colony and mammosphere formation capabilities, enhanced their motility and invasiveness and conferred them tumor formation capabilities in mouse xenografts [45]. Han et al. reported that miR-21 was found to be over-expressed in BCSCs derived from the MCF7 cell line compared to the parental cells [43]. In another study, the same research team showed that re-expression of miR-21 by MCF7 cells (MCF7/miR-21 cells) enhanced cell growth, self-renewal, clonogenicity, invasion and migration, mediated EMT conversion and regulated HIF1α expression [42]. Recent evidence from experiments with invasive and metastatic murine lines, as presented by Chi et al., indicates that miR-21 exerts its oncogenic effects via separate mechanisms, highlighting its immunosuppressive and metastatic properties. In this study, miR-21 was also capable of up-regulating genes such as NES, TRIP13 and ECM1 [46]. Interestingly, nestin (NES) is an intermediate filament protein that has been associated with BCSCs; and CD44^+^/CD24^−^ cells with high percentages of Oct4^+^ and Nestin^+^ presented with increased tumorigenicity when forming mammospheres in vitro [47].

### 2.8. miR-495

Whilst the expression of miR-495 in the majority of solid tumors has been correlated with tumor suppression, a number of tumors, such as gallbladder cancer and BC, have been described to exert tumorigenic properties [48]. Hwang-Verslues et al. found that miR-495 was the most over-expressed miRNA in a newly identified BCSC subpopulation in the MDA-MB-231 cell line, characterized by the PROCR^+^ and ESA^+^ markers, and was highly up-regulated in CD44^+^/CD24^−/low^ BCSCs derived from the SKBR3 cell line [49]. MiR-495 expression was found to be directly modulated by the E12/E47 transcription factor and miR-495 over-expression was capable of promoting oncogenesis and resistance to hypoxia by down-regulating E-cadhering and REDD1, thus contributing to stemness acquisition, invasion, and metastasis [49].

### 2.9. miR-181a and miR-181b

The miR-181 family consists of four members (miR-181a, miR-181b, miR-181c and miR-181d) that share an identical seed sequence and potentially exhibit functional redundancy [45,50]. Members of this family have been implicated in several different cancers, including breast cancer [45]. Bisso et al. reported miR-181a and miR-181b over-expression in aggressive breast cancers capable of negatively regulating the stress-sensor kinase ataxia telangiectasia mutated (ATM), and thus impairing the proper induction of DNA damage response (DDR) and the repair of DNA double-strand breaks (DSBs) [51]. Additionally, other researchers reported that miR-181 expression levels were increased when cells from BT474, MDA-MB-361 and MCF-7 cell lines were cultured as nonadherent mammospheres in comparison to two-dimensional (2D) culture conditions [50]. Furthermore, the same group revealed a novel mechanism in which TGF-β, a cytokine released by both cancer and immune cells, could induce miR-181a and miR-181b at the post-transcriptional level, subsequently leading to ATM down-regulation and enhancing the sphere-initiating stem cell-like features in breast cancer cells [50]. Finally, Iliopoulos et al. elucidated the role of miR-181b-1 in the malignant transformation of a non-transformed mammary epithelial cell line (MCF-10A) containing ER-Src [45]. The aberrantly expressed inflammatory cytokine IL-6 could activate STAT3, leading to the transcriptional activation of miR-181b-1. MiR-181b-1 directly targeted the cylindromatosis tumor suppressor gene (CYLD), leading to NF-κB activation and inducing cancer-stem-cell-like characteristics in the non-cancerous MCF-10A cell line [45].

### 2.10. miR-454-3p and -5p

miR-454 has been detected either as oncogenic or tumor-suppressive, strictly depending on the type of cancer. However, in the context of breast cancer, miR-454-5p was reported to be up-regulated and strongly associated with poor prognosis, as well as being capable of inducing EMT via targeting the FoxJ2/E-cadherin axis in MDA-MB-231 cells [52]. Ren et al. reported that miR-454-3p was amplified and over-expressed in metastatic breast cancer and capable of enhancing BCC stemness and promoting metastasis, both in vivo and in vitro. miR-454-3p could promote metastasis via the direct targeting of several negative regulators of Wnt/β-catenin signaling, such as nuclear pre-mRNA domain-containing 1A (RPRD1A), dickkopf WNT signaling pathway inhibitor (DKK3), secreted frizzled related protein 1 (SFRP1) and AXIN2 [53]. Additionally, their study revealed a positive correlation between the expression of miR-454-3p and the expression of stemness-associated genes, such as MYC, SOX2, OCT4, NANOG and SNAIL, in the MCF7 cell line [53].

### 2.11. miR-5188

miR-5188 was found to be up-regulated in breast cancer tissues, promoting breast cancer progression and functioning as a prognostic factor for the overall survival of patients. Zou et al. discovered miR-5188 to be a key player in a positive feedback loop involving miR-5188, FOXO1/β-Catenin complex and c-Jun in MCF-7 and MDA-MB-468 cells, capable of increasing stemness markers (CD44^+^ and CD133^+^) and mammosphere formation, as well as promoting metastasis, proliferation and chemoresistance in vitro and in vivo [54]. Furthermore, in this study, c-Jun was found to up-regulate miR-5188 and at the same time was up-regulated by the previous positive feedback loop [54]. Interestingly, others reported the ability of c-Jun to promote the BCSC phenotype in TNBC via the JNK/c-Jun signaling pathway and that c-Jun knockdown was able to reduce mammosphere formation and the ALDH1+ subpopulation in HCC70 and SUM149 cells [55].

### 2.12. miR-5088-5p

miR-5088-5p was recently found to be over-expressed in breast cancer and identified as an oncogenic miRNA that is involved in the promotion of breast cancer malignancy [56]. miR-5088-5p could directly target the tumor suppressor DBC2 and up-regulate MMP-2 and MMP-9, thus increasing the invasive and metastatic potential of both MCF-7 and MDA-MB-231 cells. Additionally, the over-expression of miR-5088-5p could enhance sphere formation, induce CSC markers (ALDH1, CD44, Sox2, and Oct4) and EMT markers (vimentin, Snail, Slug, and Twist). Finally, the study reported that the up-regulation of miR-5088-5p was driven, at least partially, by the hypomethylation of its promoter, which was induced by Fyn, a member of the Src family of protein tyrosine kinases [56].

### 2.13. miR-370-3p

Recent evidence suggests that miR-370-3p is highly expressed in breast cancer tissue, serum and serum exosomes, promoting BC progression, stemness and metastasis both in vitro and in vivo [57,58]. One research group reported that miR-370-3p could target and inhibit FBLN5 in MDA-MB-231 cells, thus activating the NF-κB signaling pathway and promoting the induction of EMT and stemness-related markers [57]. Furthermore, other researchers detected increased expression patterns of miR-370-3p in extracellular vesicles (EVs) derived from the highly metastatic cell lines, MDA-MB-231 and MDA-MB-436, in comparison to low-metastatic cell lines [58]. Moreover, they elucidated a novel mechanism during which EV-encapsulated miR-370-3p could down-regulate the expression of CYLD in normal breast fibroblasts (NFs), thereby inducing NF-κB signaling and activating NFs. Activated fibroblasts could, thereinafter, enhance the stemness, migration, invasion, EMT and mammosphere formation ability of low metastatic MCF-7 cells [58].

### 2.14. miR-499a-5p

MiR-499a-5p is a tumorigenic miRNA recently implicated with breast cancer. Mandal et al. found miR-499a-5p to be over-expressed in BCSC mammospheres when compared to the parental MDA-MB-231 cell line [59]. Moreover, they reported that over-expression of this miRNA could enhance the proliferation of BCCs, down-regulate the Wnt antagonist, sFRP4, and up-regulate key CSC markers, such as CD44, ALDH1, ABCC2 and ABCG2. Finally, the same team discovered that ursolic acid (UA) could function as a novel inhibitor of BCSCs by suppressing miR-499a-5p and up-regulating the Wnt antagonist, sFRP4 [59].

### 2.15. miR-221

MiR-221 is another differentially expressed miRNA in breast cancer, found to correlate with the stemness, metastasis and drug resistance of BCCs, as well as the overall survival of patients [60,61,62,63]. Ke et al. reported that miR-221 was highly expressed in basal (SUM149, HCC1954) and claudin-low (SUM159, SUM1315, MDA-MB-231) breast cancer cells, as well as ALDH1^+^ or CD44^+^/CD24^−^ BCSCs derived from luminal (MCF-7) and basal (HCC1954) cell lines [60]. Additionally, by utilizing over-expression experiments in MCF-7 cells, they found that miR-221 was capable of directly targeting ATXN1, subsequently inducing EMT-related genes (N-cadherin, Slug, Snail, Twist, Vimentin, and Occludin) and promoting the expansion of CD44^+^/CD24^−^ BCSCs [60]. Furthermore, Ye et al. found that miR-221 could directly target PTEN in the HER2-over-expressing SK-BR-3 cell line, thus maintaining the high invasiveness, metastatic potential and trastuzumab resistance of these cells [61]. Similar results were reported by another team concerning invasiveness, metastatic potential and drug resistance, but in this case, miR-221 was also capable of exerting its functions when secreted by MDA-MB-231 cells via microvesicles (MVs), and then delivered to recipient MCF-7 cells [62]. Moreover, in another study, miR-221 was found to be up-regulated in T47D BCSCs and capable of directly targeting DNMT3b, thus increasing mammosphere formation and stem cell marker (Nanog, Oct3/4) expression [63].

### 2.16. mir-301a-5p

MiR-301a-5p has been described as an oncogenic miRNA in breast cancer as well as several other cancers, such as pancreatic, gastric, hepatocellular and colorectal [64,65]. In studies regarding TNBC patients, high mir-301a expression was found to be associated with increased tumor size, lymph node metastasis and poor overall survival [65,66]. Interestingly, Lettlova et al. observed miR-301a-5p to be highly expressed in stem-like cells from the MCF-7, ZR-751 and T47D cell lines, when cultured in mammosphere conditions [64]. The same researchers reported that the over-expression of miR-301a-5p in MCF-7 cells implanted in nude mice inhibited ER signaling and up-regulated stem-cell, EMT and metastasis markers such as CD44, ALDH1, ABCG2, Vimentin, ZEB1, ZEB2, HER2 and VEGFA [64].

### 2.17. miR-520b

MiR-520b was found to be up-regulated in BC tissues and cell lines and to function as an indicator of poor prognosis. Moreover, Zhang et al. detected miR-520b up-regulation in CD44^+^, CD133^+^ and ALDH1^+^ cells, derived from both MCF-7 and MDA-MB-231 cell lines, when compared to the corresponding CD44^−^, CD133^−^ and ALDH1^−^ cells, indicating a selectivity for BCSCs [67]. The miR-520b could promote the stemness of cancer stem cells, as indicated by an increase in gene expression associated with sphere formation, migration and stemness (N-cadherin, Vimentin, Snail1 and ZEB1) in both MCF-7 and MDA-MB-231 cell lines. Finally, they elucidated a potential mechanism guiding the aforementioned stem-cell related functions that involved the direct targeting of LATS2 by miR-520b and subsequent activation of the Hippo/YAP signaling pathway [67].

### 2.18. miR-31

A pro-oncogenic role for miR-31 in breast cancer has been proposed, since it was found enriched in mammary stem cells (MaSC) and breast tumors, and appears to be essential for maintaining mammary tumor stem cells (CD24^+^/CD90^+^) in the MMTV-PyVT mice model [68]. Moreover, the depletion of miR-31 in MMTV-PyVT mice led to compromised tumor growth, a reduced number of cancer stem cells (CD24^+^/CD90^+^), decreased tumor-initiating ability and impaired metastasis to the lung. Finally, miR-31 was found to exert various functions via the activation of Wnt/β-catenin signaling by directly targeting Wnt antagonists, such as Dkk1, Axin1 and Gsk3β, as well as the repression of the TGF-β signaling potentially by targeting Smad3 and Smad4 in the mammary epithelium [68].

### 2.19. mir-1290

MiR-1290 is a novel and potentially oncogenic miRNA that was found to be up-regulated in HER2^+^, TNBC tumors and tumor extracellular vesicles (EVs). It was also correlated with brain metastasis, as well as worse metastasis-free survival and brain metastasis-free survival [69,70]. Sirkisoon et al. reported that miR-1290 enclosed within BC EVs was capable of activating astrocytes in the brain metastatic microenvironment via the suppression of FOXA2 and subsequent up-regulation of ciliary neurotrophic factor (CNTF) [69]. Interestingly, conditioned medium from miR-1290-activated astrocytes was capable of promoting mammosphere formation, while miR-1290 over-expression in SKBR3 cells significantly enriched CD44^+^/CD24^−^ BCSCs cells [70].

### 2.20. miR-29a

The miR-29 family consists of three mature members, miR-29a, miR-29b, and miR-29c. Two members, namely miR-29b and mir-29c, seem to possess tumor suppressor capabilities [71,72]. Interestingly, Wu et al. found miR-29a to be up-regulated in human BC tissues compared to distal healthy tissues, as well as in BCSCs and spheroid cells from the MCF-7 cell line, as compared to MCF-7 cells, and in the aggressive TNBC cell line MDA-MB-231 [73]. Mir-29a was found to be essential for the highly migrative and invasive ability of aggressive breast cancer cells and BSCSs, as well as EMT promotion in MCF-7 cells in vitro, all of which were mediated by the direct targeting of SUV420H2 methyltransferase via miR-29a and the subsequent down-regulation of H4K20me3, whichpromoted EGR1 and CTGF over-expression [73].

### 2.21. miR-22

MiR-22 potentially possesses a dual function in cancers, by either inhibiting or promoting cancer [74]. The miR-22 and its target, TIP60, were associated with EMT in breast cancer, and the miR-22(high)/TIP60(low) axis was suggested to function as an indicator of breast cancer progression and poor overall survival [75]. In the context of CSCs generation and breast tumor formation, Song et al. examined the effect of miR-22 over-expression on the non-tumorigenic human breast epithelial cell line MCF-10A and the non-metastatic breast cancer cell line MCF-7 [76]. They observed that miR-22 could directly inhibit TET and subsequently epigenetically silence the anti-metastatic miR-200, thus increasing EMT, stemness and metastasis in both cell lines [76]. Interestingly, another group found miR-200 to be down-regulated in BCSCs (CD44^+^/CD24^−/low^) derived from human breast cancer samples [77]. These researchers reported that miR-200 could directly target the regulator of stem cell self-renewal BMI1 and suppress the proliferation and self-renewal of BCSCs both in vitro and in vivo. Despite the aforementioned oncogenic properties of miR-22, several reports can be found in the literature concerning this miRNA’s tumor suppressive functions in BC [78,79].

### 2.22. miR-146a

MiR-146a was found to be up-regulated in CD44^+^CD24^−^ BCSCs from human breast cancer samples and in cisplatin-resistant MCF-7 cells [77,80]. Wang et. al. observed that miR-146a, along with KLF8, was significantly up-regulated in the invasive cancer cell lines, MDA-MB-231, Hs578T and BT-549, but not the non-invasive ones, namely MCF-7 and T47-D [81]. Furthermore, they discovered that KLF8 was responsible for up-regulating miR-146a, which in turn activated Notch signaling by targeting a known Notch inhibitor, NUMB, in MCF-10A cells and potentially in BCSCs. In vitro and in vivo experiments verified that this mechanism was sufficient for promoting the malignant transformation of MCF-10A cells and inducing pro-tumorigenic mammary stem cells [81]. Another research group showed that miR-146a played a crucial role in the self-renewal of BCSCs and that miR-146a expression in the SUM159, triple-negative breast cancer cell line affected mammosphere formation, tumor-initiating cell (TIC) frequency and drug resistance [82].

The effect, the targets and the associated cell lines of each miRNA discussed in this section are presented in Table 1, below.

## 3. Discussion

Cancer stem cells (CSCs) play an essential role in tumorigenesis. In the context of breast cancer (BC), accumulating evidence links breast cancer stem cells (BCSCs) with processes such as BC formation, progression and metastasis. Although their roles seem to be crucial, the origins of BCSCs have not yet been elucidated, and there are a number of hypotheses around their identification.

A widely accepted suggestion is that BCSCs originate from mammary stem cells (MaSCs) or progenitor cells, as they share similar phenotypic features. Additionally, the long-lived nature of stem cells makes them more susceptible to acquiring and accumulating genetic mutations, ultimately leading to oncogenic transformation [83]. In support of this hypothesis, several miRNAs, such as miR-21, -22, -31, -146a and -181b-1, are capable of promoting and maintaining the malignant transformation of MaSCs, which seem to obtain a BCSC phenotype and an increased tumor-initiating ability.

On the other hand, another hypothesis proposes that BCSCs are derived from non-stem-differentiated cancer or pro-tumorigenic cells that acquire mutations, thereby activating stemness-related pathways and leading them to de-differentiation [84,85]. According to this hypothesis, the aggressive features of malignancies are not derived from their existing content of CSCs, but from their ability to generate de novo CSCs from non-CSC populations. In support of this hypothesis, miRNAs such as miR-21, -22, -24, -155, -210, -370-3p, -499a-5p, -5188, -5088-5p and many others, have been found capable of regulating the stemness of several cancer cell lines.

**Figure 2:**
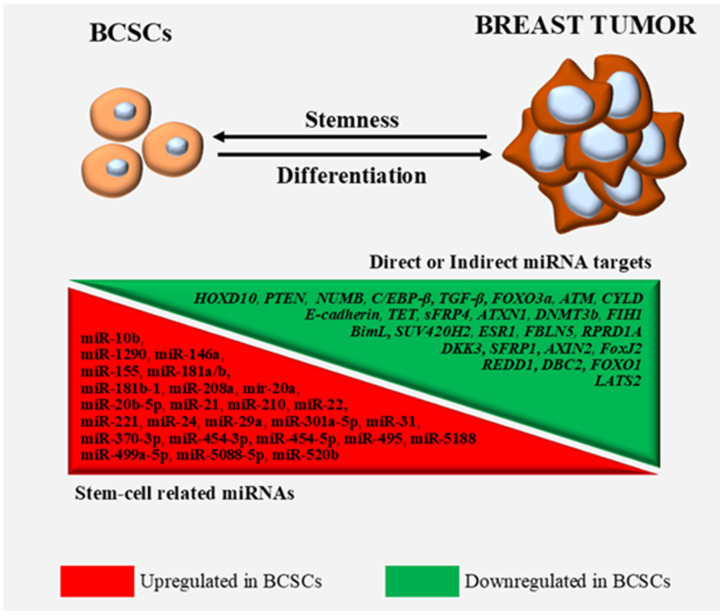
MiRNAs involved in BCSC (and stem-like BCC) processes and their targets.

Despite the different theories regarding the origins of CSCs, solid tumors are dynamic entities characterized by heterogeneity and hierarchy [9,86,87] that constantly interact with, shape and are shaped by their microenvironment [88]. The formation and clonal expansion of BCSCs and/or the acquisition of stem-like characteristics via BCCs (Figure 2) seem to modulate the outcome with regards to immuno-evasion, progression, metastasis and therapy resistance. For this reason, there is a need, in our opinion, for a unified strategy to identify the existence of BCSCs within tumors and different cell lines, the exact relation of BCSCs with stem-like BCCs, the specific functions and the contributions of these cells in cancer formation, progression and metastasis.

EMT is a pivotal step in invasion and cancer metastasis since epithelial cells, which are immotile and tightly adherent to the surrounding matrix and to one another, acquire a mesenchymal phenotype [89].

A strong correlation between epithelial-to-mesenchymal transition (EMT) and the stem-cell phenotype of BCCs or the stemness of BCSCs, is evident in several studies reviewed here. In this context, we observed known mesenchymal markers (N-cadherin, Vimentin, MMP2, MMP9 and CTGF) and EMT-related transcriptional factors (Snail, Slug, Zeb1, Zeb2, Twist, KLF8, SOX2, HIF1α and EGR1) to be strongly induced in stem-like BCCs and/or BCSCs, with an apparent implication of several miRNAs [90,91,92]. Although there seems to be a positive correlation between EMT and stemness, this does not appear to be an absolute rule since a number of studies have shown that reversion of EMT can be accompanied by an induction of cancer stem cell properties and an increase in the stemness of CSCs [93,94]. In our opinion, such conflicting observations highlight the complex interactions and events guiding cancer progression and metastasis.

It is worth mentioning that, there seem to be conflicting reports in the literature concerning the oncogenic or tumor-suppressive role of a number of miRNAs in BC and BCSCs [95,96,97]. For example, in several studies, miR-9 is presented as an oncogene associated with increased invasiveness and the acquisition of stem-like traits of BCCs, while in other studies miR-9 is presented as a tumor suppressor capable of inhibiting BCC proliferation, inducing apoptosis and increasing the responsiveness to drug therapies [95]. Accordingly, in one study, miR-195-5p over-expression is shown to inhibit the colony formation and cell migration of BCCs, while in another study, miR-195-5p, contained in EVs induced by chemotherapy, is capable of increasing the expression of known stemness-associated genes, thus stimulating stem-like characteristics [96,97]. Different BC subtypes, cancer staging and/or experimental conditions could offer an explanation for the observation of contradictory functions, but for the purposes of this review, the aforementioned miRNAs, together with some others, presenting dual functions in BC, were not included.

Therapy resistance is a critical event with regard to the management of patients suffering from BC. Several commercial therapies and different approaches based on the intrinsic subtypes of BC are available, but patients continue to experience relapse and the long-term mortality levels remain high [98]. There is enough evidence to associate BCSCs with therapy resistance [9,10,98]. In our research, we found that miRNAs, such as miR-21, miR-24, miR-155, miR-210, miR-221 and miR-5188, could represent links between cancer stemness, EMT conversion and resistance to therapies. Finally, one miRNA, namely miR-20a, appeared to be implicated in immunoevasion, and is capable of assisting the escape of BCSCs from innate immunity, subsequently leading to distant metastasis.

The involvement of BCSCs in BC is becoming increasingly evident and the regulation of these cells by a variety of miRNAs, together with other genetic and epigenetic factors, create a new area of research interest. Several existing databases, such as “miRBase” and “miRDB”, have been created in order to aid researchers in the investigation of miRNAs [99,100]. However, we firmly believe that this effort could be enhanced by constructing CSC-specific miRNA databases, providing data from the miRNA profiling of CSCs isolated from bulk cancer cell populations in primary tumor samples and different cancer cell lines.

A deeper understanding of the relationship between CSCs and miRNAs is imperative for the development and application of more effective anti-cancer therapies. Currently, the utility of miRNAs in breast cancer research is limited to predictive and prognostic purposes, with several prospective clinical trials reaching phases II and III [101]. On the other hand, clinical trials focused on the therapeutic applications of miRNAs are relatively limited, with only a few reaching phase I and even fewer reaching phase II [102]. Interestingly, there seem to be no clinical trials involving miRNAs and BCSCs.

In conclusion, miRNAs are shown to be actively implicated in cancer progression, EMT, and metastasis via the regulation of oncogene and onco-suppressor expression and the promotion of the BCSC phenotype. Therefore, miRNAs enhance the malignant behavior of breast tumors, increasing tumor heterogeneity and resistance to therapies. Targeting molecules and regulators involved in stemness-related features of cancer cells and cancer stem cells, may provide a solid basis of perspective therapeutic strategies, aiming to eliminate surviving cancer cells, thus preventing recurrence and improving the long-term survival of breast cancer patients.

## Figures and Tables

**Table 1:** The involvement of miRNAs in BCSC- (and stem-like BCC)-related processes.

miRNA	Effect	Targets	Implicated Cells & Cell Lines	References
miR-10b	Invasion, Migration	*HOXD10*	MDA-MB-231, SUM149, SUM159	[40]
	Self-renewal, Stemness, EMT	*PTEN*	MCF-7, SKBR-3, MDA-MB-231	[41]
miR-1290	Mammosphere formation, TME modulation, Brain metastasis,	*-*	SKBR3	[69,70]
miR-146a	Malignant transformation, Pro-tumorigenic mammary stem cell formation	*NUMB*	MCF-10A	[81]
	Self-renewal, Mammosphere formation, Tumor initiating cells frequency, Drug resistance	*-*	SUM159	[82]
miR-155	Sphere growth, Stem-cell formation, Doxorubicinol resistance	*-*	MDA-MB-231	[27]
	Stem-like characteristics, EMT-related resistance, Migration	*C/EBP-β*, *TGF-β*, *FOXO3a*	MCF-7, MDA-MB-231	[28]
miR-181a/b	DDR impairment	*ATM*	MDA-MB-231, SUM159PT	[51]
	Mammosphere formation, Stem-like characteristics	*ATM*	BT474, MDA-MB-361, MCF7	[50]
miR-181b-1	Malignant transformation, BCSC formation	*CYLD*	MCF-10A	[45]
miR-208a	BCSC phenotype, Increased BCSC proportion	*-*	MDA-MB-231, ER^+^ ZR75-1	[29]
mir-20a	BCSC resistance to NK cell cytotoxicity, Lung metastasis	*MICA*, *MICB*	Primary BCSCs	[38]
miR-20b-5p	BCSC proliferation, BCSCs inhibition of apoptosis	*CCND1*, *E2F1*	T47D-BCSCs	[39]
miR-21	Chemoresistance	-	MDA-MB-468	[44]
	BCSC-like characteristics, Colony and mammosphere formation, Motility, Invasiveness, Tumor formation capabilities	*PTEN*	MCF-10A	[45]
	Cell growth, Self-renewal, Clonogenicity, Invasion, Migration, EMT	-	MCF-7	[42,43]
miR-210	Radioresistance	-	MCF-7, MDA-MB-231	[31]
	Proliferation, Self-renewal, Invasion, Migration	*E-cadherin*	MCF-7	[32]
miR-22	BCSC promotion, Tumor formation, Stemness, EMT, Metastasis	*TET*	MCF-7, MCF-10A	[76]
miR-221	BCSC promotion, EMT,	*ATXN1*	MCF-7	[60]
	Invasion, Metastasis, Chemoresistance	*PTEN*	SK-BR-3	[61]
	Invasion, Metastasis, Chemoresistance	*PTEN*	MCF-7, MDA-MB-231	[62]
	BCSC phenotype, Mammosphere formation	*DNMT3b*	T47D	[63]
miR-24	Mammosphere formation, Stemness, Adaptive hypoxia response, Cisplatin resistance	*FIH1*, *bimL*	MCF-7, T47D, MDA-MB-231	[34]
miR-29a	Invasion, Migration, EMT	*SUV420H2*	MCF-7, MDA-MB-231	[73]
miR-301a-5p	BCSC phenotype, EMT, Metastasis	*ESR1*	MCF-7	[64]
miR-31	Tumor formation, BCSC promotion, Lung metastasis	*Dkk1*, *Axin1*, *Gsk3β*, *Smad3*, *Smad4*	Mammary epithelial cells, CD24^+^/CD90^+^ BCSCs from PyVT tumors	[68]
miR-370-3p	Stemness, EMT	*FBLN5*	MDA-MB-231	[57]
	Stemness, Migration, Invasion, EMT, Mammosphere formation	*CYLD*	MCF-7, MDA-MB-231, MDA-MB-436, Primary NFs	[58]
miR-454-3p	Stemness, Metastasis	*RPRD1A*, *DKK3*, *SFRP1*, *AXIN2*	MCF-7, MDA-MB-231	[53]
miR-454-5p	EMT	*FoxJ2*	MDA-MB-231	[52]
miR-495	Stemness, Invasion, Metastasis, Early relapse	*E-cadherin*, *REDD1*	MDA-MB-231, SKBR3	[49]
miR-499a-5p	BCSC phenotype, Mammosphere formation, Proliferation	*sFRP4*	MDA-MB-231	[59]
miR-5088-5p	BCSC phenotype, Mammosphere formation, EMT, Invasion, Metastasis	*DBC2*	MCF-7, MDA-MB-231	[56]
miR-5188	Stemness, Mammosphere formation, Proliferation, Metastasis, Chemoresistance	*FOXO1*	MCF-7, MDA-MB-468	[54]
miR-520b	Stemness, Sphere formation, Migration	*LATS2*	MCF-7, MDA-MB-231	[67]

## Data Availability

Not applicable.
